# Multisystem Inflammatory Syndrome therapies in children (MISTIC): A randomized trial^☆^

**DOI:** 10.1016/j.conctc.2023.101060

**Published:** 2023-01-20

**Authors:** Sonia Jain, Feng He, Kiana Brown, Jane C. Burns, Adriana H. Tremoulet

**Affiliations:** a Biostatistics Research Center, Herbert Wertheim School of Public Health and Human Longevity Science, University of California at San Diego, USA; b Department of Pediatrics, UCSD School of Medicine/Rady Children's Hospital San Diego, 9500 Gilman Dr, Mail Code 0641, La Jolla, CA, 92093-061, USA

**Keywords:** Multisystem inflammatory syndrome in children (MIS-C), Infliximab, Anakinra, Steroids, snSMART, Randomized clinical trial, IVIG, Intravenous immunoglobulin, snSMART, small N Sequential Multiple Assignment Randomized Trial

## Abstract

**Background:**

Multisystem Inflammatory Syndrome in Children (MIS-C), which occurs 2–6 weeks after initial exposure to SARS-CoV-2, was first identified in early 2020 when patients presented with fever and significant inflammation, often requiring management in the intensive care unit. To date, there has been no clinical trial to determine the most effective treatment. This study compares anti-inflammatory treatments that were selected based on current treatments for Kawasaki disease, a coronary artery vasculitis that shares many clinical features with MIS-C.

**Methods:**

This randomized, comparative effectiveness trial of children with MIS-C uses the small N Sequential Multiple Assignment Randomized Trial (snSMART) design for rare diseases to compare multiple therapies within an individual. Study participants were treated first with intravenous immunoglobulin (IVIG), and if needed, subjects were then randomized to one of three additional treatments (steroids, anakinra, or infliximab). Participants were re-randomized to remaining treatments if they did not demonstrate clinical improvement.

**Conclusion:**

This trial continues to enroll eligible participants to determine the most effective therapies in addition to IVIG and best order in which to use them to treat MIS-C.

**Trial Registration:**

NCT04898231.

## 1 Introduction

Multisystem Inflammatory Syndrome in Children (MIS-C), a post-inflammatory syndrome in children following infection with SARS-CoV-2, was first described in April 2020, at the onset of COVID-19 pandemic [1]. The clinical presentation of MIS-C shares many features with Kawasaki disease (KD), a coronary artery vasculitis that presents with fever, rash, conjunctival injection, mucocutaneous erythema, and cervical lymphadenopathy. Given these clinical similarities, physicians treated the majority of MIS-C patients with intravenous immunoglobulin (IVIG), the standard of care for KD patients [2,3]. Some MIS-C patients did not improve with IVIG alone and were treated with steroids, anakinra (IL-1 blockade), or infliximab (TNFα blockade), all anti-inflammatory therapies that have been successfully used in the treatment of children with KD [[4], [5], [6], [7], [8], [9]]. There has been no randomized clinical trial to determine the effectiveness of these therapies. Therefore, we designed a randomized, comparative effectiveness trial to determine the best combination of anti-inflammatory therapies and the order in which they should be given in the treatment of MIS-C.

## 2 Rationale for therapeutic choices in this study

There are several lines of evidence that support the use of IVIG, steroids, anakinra and infliximab in KD, and thus these were used as the rationale for using these same therapies to treat MIS-C patients. Over the last two years of the pandemic, observational studies have demonstrated the effectiveness of one or more of these therapies, thus providing further support for their use in the clinical care of these patients [[10], [11], [12], [13]].

In KD, IVIG boosts the host's anti-inflammatory response by stimulating tolerogenic myeloid dendritic cells to secrete the immunosuppressive cytokine IL-10, which inhibits pro-inflammatory cytokine release from most immune cells [14]. Recently, Zhu et al. have shown that IVIG is a potent activator of neutrophil cell death in KD and MIS-C patients via PI3K and NADPH oxidase [15]. The observation that MIS-C is mediated by a robust innate immune response with activated neutrophils secreting IL-1b provided a strong rationale for use of IVIG as first-line therapy.

Steroids have been extensively used as an anti-inflammatory therapy in several vasculitides in childhood and are used in conjunction with IVIG by many centers for KD patients with coronary artery abnormalities (CAA) or persistent fever [4]. Coronary artery dilation can be a feature of MIS-C and thus it was logical to choose steroids as a therapy given that steroids in combination with IVIG have been shown to significantly reduce the incidence of CAA in KD patients when compared to treatment with IVIG alone [4].

Anakinra, a recombinant IL-1 receptor antagonist, competitively inhibits IL-1 binding to the IL-1 type 1 receptor. Genes in the IL-1 pathway are upregulated in the peripheral blood of KD patients during the acute phase of illness [16]. Anakinra blocks arterial inflammation in a murine model of KD [17]. A Phase I/IIa study of anakinra in KD patients with CAA demonstrated the safety and sustained serum concentration of IV anakinra in patients treated with doses up to 11 mg/kg/day [18]. An additional clinical trial of anakinra for persistent fever in KD patients also demonstrated the safety and tolerability of this biologic [6]. Given the familiarity with IL-1 blockade in KD and the demonstration that IL-1b played a major role in the inflammation in MIS-C, anakinra was used in the treatment of MIS-C patients early in the pandemic [11].

Infliximab is a monoclonal antibody that binds to and reduces levels of circulating tumor necrosis factor alpha (TNFα). Given that TNFα is increased in acute KD and highest in children with coronary artery aneurysms, infliximab has been used to treat KD [19,20]. A Phase III randomized trial of infliximab in children with IVIG-resistant KD demonstrated the safety of infliximab. Infliximab reduced inflammation more rapidly than a second dose of IVIG [5]. A recent randomized, multicenter, comparative effectiveness trial of infliximab vs. 2nd IVIG in IVIG-resistant KD in the US (KIDCARE) further demonstrated the safety and efficacy of infliximab and revealed the increased risk of hemolytic anemia with increasing doses of IVIG [21]. Infliximab was recently approved for the treatment of refractory KD in Japan and a multicenter, prospective, open-label study in Japan further demonstrated the safety of infliximab in KD patients [8]. Thus, due to the use of infliximab in refractory KD patients, physicians started treating complicated MIS-C patients with infliximab [12].

Given the similarities between KD and MIS-C, therapies that were safe and effective for treatment of KD were also used for children with MIS-C. In the absence of clinical trial data for this new condition, centers around the globe reached for some combination of these anti-inflammatory to treat MIS-C patients [22]. However, a clinical trial was urgently needed to evaluate which anti-inflammatory regimen was most effective. Thus, this trial was designed and launched to fill this knowledge gap.

## 3 Objectives

The main goal of this study is to determine the anti-inflammatory therapy from first randomization with the lowest rate of second randomization in the treatment of MIS-C. This study will also evaluate the best order in which the therapies should be given to achieve the greatest therapeutic effect.

## 4 Study design

This study is a randomized comparative effectiveness trial of children with MIS-C using the patient-centered “small N Sequential Multiple Assignment Randomized Trial (snSMART) designed for rare disease trials as it efficiently evaluates multiple treatments within an individual [[23], [24], [25]].

**Fig. 1:**
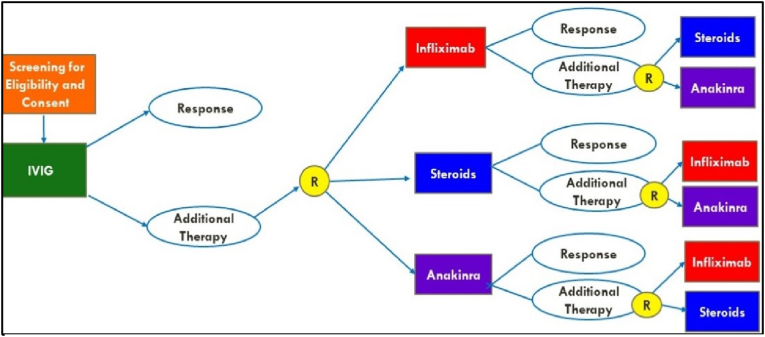
**snSMART study overview for MIS-C patients**. Study drugs administered as follows: Infliximab: 10 mg/kg IV dose once; Steroids: 2 mg/kg IV divided BID (max 60 mg) with steroid taper for 2–3 weeks post-discharge; Anakinra: Up to 10 mg/kg/day (max 200 mg every 6 h) with taper and discontinuation prior to discharge. *Color to be used.* (For interpretation of the references to color in this figure legend, the reader is referred to the Web version of this article.)

All subjects will be treated with IVIG at the standard dose of 2 g/kg over 12 h (max 100g; for obese patients, dose based on ideal body weight). Subjects will subsequently be randomized to receive additional anti-inflammatory therapy of steroids (2 mg/kg/day methylprednisolone IV divided q12 h; max 60 mg/day), anakinra (up to 10 mg/kg/day IV with max 200 mg q6h) or infliximab (10 mg/kg IV once over 2 h) if needed. This study allows for re-randomization to one of the two remaining arms if clinically warranted (See Fig. 1)

Randomization to additional therapy is at the discretion of the treating physician (but not required) for patients who meet one or more of the criteria listed in Table 1, including fever after completion of IVIG, persistent or worsening inflammation, and/or persistent or worsening cardiac dysfunction. The schedule of all tests and laboratory studies is shown in Table 2.

**Table 1:** Criteria for randomization to additional therapy after completion of IVIG infusion.

1 Fever (38 °C) ≥12 h after completion of IVIG 2 Fever (38 °C) ≥12 h after completion of IVIG and initial randomized treatment arm 3 Persistent or worsening inflammation, including but not limited to: a CRP increase ≥20% from baseline >24 h after completion of IVIG alone or IVIG and initial randomized treatment arm 4 Organ dysfunction, including but not limited to: a.Coronary artery Z score ≥2.5 b. Left ventricular dysfunction (ejection fraction ≤55%) c. Valvulitis d. Hypotension e. Need for inotropic support 5 Persistent or worsening organ dysfunction, including but not limited to: a Worsening coronary artery Z score b. Worsening left ventricular dysfunction c. Worsening valvulitis d. iew need for, or increase in, inotropic support

**Table 2:** Schedule of study data and sample collection The schedule of all tests and laboratory studies is depicted in the following table.

Time point	Baseline (prior to any therapy)	12 h after IVIG completed (prior to additional tx)	12 h after first randomized therapy completed (prior to add'l therapy)	12 h after second randomized therapy (or just prior to discharge if no further randomization)	2 weeks after discharge
Visit Window		+1 day	+1 day	+1 day	±10 days
Informed Consent/Assent	X				
Inclusion/Exclusion Criteria	X				
Weight and Height Measurements	X				X
Medical history	X				
Demographics	X				
Concomitant Meds	X	X	X	X	X
Physical Exam	X	X	X	X	X
Urine Pregnancy Test^a^	X				
Clinical Laboratory tests (e.g. CBC, CRP)^b^	X	X	X	X	X
Research Study Samples^c^	X	X	X	X	X
Echocardiogram^b^	X	X	X	X	X
Adverse events assessment	X	X	X	X	X

a For females of child-bearing potential.

b Most closely associated with study lab draw if available.

c Over a 30-day period, the total volume of research samples will not exceed 2 ml/kg. Research samples volumes will be adjusted as needed depending on patient's weight. Blood samples are limited to 8.5 ml of blood distributed between red top tubes for serum, purple top tubes for plasma, and PAX-gene tubes for whole blood RNA.

**Fig. 2:**
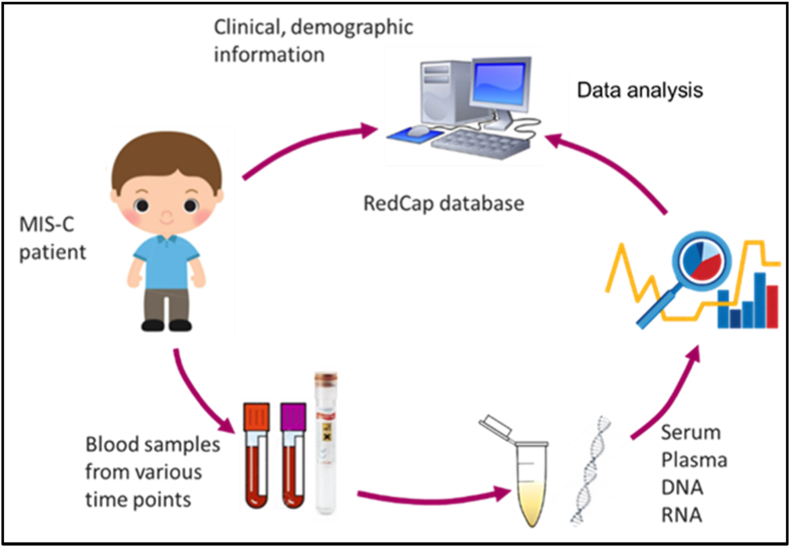
**Sample collection process***Color to be used.* (For interpretation of the references to color in this figure legend, the reader is referred to the Web version of this article.)

Clinical, laboratory, and echocardiographic data will be obtained as part of the routine care of the patient (e.g. hematology and chemistry results, medications) and stored in a HIPPAA-secure REDCap Database. Plasma, serum, whole blood RNA and DNA samples will be collected at various time points for future analysis (See Fig. 2).

Echocardiography will include measurements of (1) the coronary arteries: internal lumen of the proximal right and proximal left anterior descending coronary arteries, normalized to body surface area and expressed as z-scores and American Heart Association classification schema for coronary artery aneurysms and (2) ventricular function, valves and pericardial effusion, including quantitative assessment of left ventricular (LV) size and systolic function (LV end-diastolic volume, ejection fraction, and longitudinal strain), presence and degree of mitral and aortic regurgitation by standard color flow mapping and pulsed Doppler techniques, and parameters of LV diastolic function with tissue Doppler imaging and MV inflow. All quantitative measures will be measured on three consecutive beats and values will be averaged.

## 5 Inclusion and exclusion criteria

Patients will be eligible to participate if they meet the CDC criteria for MIS-C. This includes fever, at least two body systems involved, predominantly cardiovascular, mucocutaneous, or gastrointestinal, and have no other leading diagnosis. In addition, proof of exposure to SARS-CoV-2, usually by positive IgG to SARS-CoV-2 nucleocapsid, will be needed. Parent or legal guardian must be able and willing to provide informed consent and subject willing and able to provide assent when appropriate.

Patients will be excluded if they have an immunodeficiency, a medical condition that prevents them from receiving one or more of the study drugs, or have received any of the study drugs prior to enrollment.

## 6 Statistical methods

### 6.1 Statistical analysis plan

Since this is a superiority, snSMART study design, an intent-to-treat (ITT) approach will be used to analyze the data regarding patient outcomes. A per protocol analysis of patients completing the protocol will be performed as a sensitivity analysis analogous to the ITT analyses. Results will be reported as point estimates (odds ratios or mean differences across groups, as appropriate) and interval estimates (95% confidence intervals). All tests of significance for the secondary outcomes will be 2-sided and Hochberg adjustments will be made for multiple comparisons. A p-value of 0.05 will be considered statistically significant unless otherwise specified. Statistical analyses will be conducted using the statistical software R 3.6.3 (www.rproject.org). and rjags. Demographic and baseline characteristics will be compared among the study arms using Fisher's exact test for categorical variables, and ANOVA/pairwise two-sample t-tests for continuous variables. Appropriate non-parametric alternatives will be considered if parametric assumptions fail.

### 6.2 Sample size and power

We project enrollment of 180 children with MIS-C. We anticipate that 50 (27%) children will improve with IVIG alone, while 130 (73%) will require additional treatment post-IVIG resulting in randomization across the three interventions over the study period. With a worst-case attrition rate of 17%, we will achieve 108 evaluable subjects for the snSMART design. All sample size calculations were performed using the snSMART Sample Size App [https://umich-biostatistics.shinyapps.io/snsmart _sample_ size_app/]. This two-stage design uses a Bayesian joint stage model for estimating the response rates of each individual treatment in a three-arm snSMART design. The approach distinguishes the best treatment from the second-best treatment using the Bayesian joint stage model. We require approximately 36 subjects per arm (108 in total for three agents) at the initial randomization. The probability of successfully identifying the best treatment is 0.85, when the difference of response rates between the best and second-best treatment is at least 0.25 and the response rate of the best treatment is 0.75. We conducted sensitivity analyses for the power using the joint-stage regression model (JSRM) method of Chao et al. (2020) [25] that used Dunnett's approach under generalized estimating equations (GEE) and obtained similar results for approximately 85% power and alpha = 0.05. Therefore, an anticipated sample size of approximately 108 evaluable subjects should provide reasonable evidence for a treatment effect in this rare disease setting. Note that if attrition is slightly worse at 25%, then we would require an overall N of 144, in which case we would still be well-powered with this sample size.

### 6.3 Analysis of the primary outcome for aim 1

Comparisons between the three treatment arms with respect to the primary endpoint of best clinical response rate will be conducted using a Fisher's exact test for proportions. Differences in the rates between the multiple arms, along with the odds ratio (OR) and their 95% confidence intervals will be reported. A Bayesian Joint Stage Model will be considered, which uses all data from both randomized and re-randomized subjects in estimation and inference, using “linkage parameters” to connect the data from each randomization. This approach provides an unbiased and efficient estimation of the treatment effects and dynamic treatment regimens. Also, as a sensitivity analysis, a multivariable joint-stage GEE regression model will be performed to study the association between clinical, biomarker and demographic factors and intervention arm, adjusting for baseline demographic, stratification factors, and clinical characteristics. Variables significantly associated with both treatment group and outcome (p < 0.10) will be included in a multivariable model as covariates.

### 6.4 Comparison to IVIG alone

The baseline characteristics and outcomes of subjects treated only with IVIG will be compared to those randomized to receive infliximab, anakinra or steroids.

## 7 Conclusion

This study continues to enroll eligible patients to determine the most effective treatment regimen in addition to IVIG and the best order of therapies for MIS-C. Recently, this study has expanded from its initial enrollment center at Rady Children's Hospital San Diego, to also include enrollment at Children's Hospital Michigan. Additional sites will be considered if MIS-C persists in sufficient numbers.

## Author contributions

**Sonia Jain:** Methodology, Formal Analysis, Writing-original draft preparation.

**Feng He:** Formal Analysis.

**Kiana Brown**: Data curation, Writing-original draft preparation, review and editing.

**Jane C. Burns**: Conception, Writing-review and editing, Supervision.

**Adriana H. Tremoulet:** Conception, Writing-review and editing, Supervision.

## Declaration of competing interests

The authors declare that they have no known competing financial interests or personal relationships that could have appeared to influence the work reported in this paper.
